# Detection of influenza virus in urban wastewater during the season 2022/2023 in Sicily, Italy

**DOI:** 10.3389/fpubh.2024.1383536

**Published:** 2024-07-23

**Authors:** Carmelo Massimo Maida, Walter Mazzucco, Walter Priano, Roberta Palermo, Giorgio Graziano, Claudio Costantino, Arianna Russo, Gina Andolina, Isabella Restivo, Viviana Giangreco, Francesca Rita Iaia, Arianna Santino, Rita Li Muli, Valeria Guzzetta, Francesco Vitale, Fabio Tramuto

**Affiliations:** ^1^Department of Health Promotion, Mother and Child Care, Internal Medicine and Medical Specialties “G. D’Alessandro”, University of Palermo, Palermo, Italy; ^2^Clinical Epidemiology Unit, Regional Reference Laboratory of Western Sicily for the Emergence of COVID-19, University Hospital “P. Giaccone”, Palermo, Italy

**Keywords:** wastewater, surveillance, wastewater-based epidemiology, influenza viruses, influenza season

## Abstract

**Introduction:**

Seasonal influenza generally represents an underestimated public health problem with significant socioeconomic implications. Monitoring and detecting influenza epidemics are important tasks that require integrated strategies. Wastewater-based epidemiology (WBE) is an emerging field that uses wastewater data to monitor the spread of disease and assess the health of a community. It can represent an integrative surveillance tool for better understanding the epidemiology of influenza and prevention strategies in public health.

**Methods:**

We conducted a study that detected the presence of Influenza virus RNA using a wastewater-based approach. Samples were collected from five wastewater treatment plants in five different municipalities, serving a cumulative population of 555,673 Sicilian inhabitants in Italy. We used the RT-qPCR test to compare the combined weekly average of Influenza A and B viral RNA in wastewater samples with the average weekly incidence of Influenza-like illness (ILI) obtained from the Italian national Influenza surveillance system. We also compared the number of positive Influenza swabs with the viral RNA loads detected from wastewater. Our study investigated 189 wastewater samples.

**Results:**

Cumulative ILI cases substantially overlapped with the Influenza RNA load from wastewater samples. Influenza viral RNA trends in wastewater samples were similar to the rise of ILI cases in the population. Therefore, wastewater surveillance confirmed the co-circulation of Influenza A and B viruses during the season 2022/2023, with a similar trend to that reported for the weekly clinically confirmed cases.

**Conclusion:**

Wastewater-based epidemiology does not replace traditional epidemiological surveillance methods, such as laboratory testing of samples from infected individuals. However, it can be a valuable complement to obtaining additional information on the incidence of influenza in the population and preventing its spread.

## 1 Introduction

Influenza is a viral acute respiratory infection with high morbidity and mortality in humans, especially in specific groups such as children and older adults, posing a constant threat to global public health because of recurring seasonal epidemics and irregularly occurring pandemics (1–3). The burden of this disease can vary widely, being determined by several factors, including the characteristics of circulating viruses, the timing of the season, the environmental temperature, how well the available vaccine is working to protect against illness, and how many people got vaccinated (4, 5). The Centers for Disease Control and Prevention (CDC) estimated that influenza has resulted in 9 million–41 million illnesses, 140,000–710,000 hospitalizations, and 12,000–52,000 deaths annually between 2010 and 2020 in the United States (6). Seasonal influenza epidemics have substantially contributed to the worldwide annual mortality rate, particularly among the older adult 65 years and over. In Italy, a mortality rate of 10.7 per 1,000 inhabitants was observed in the winter season of 2014/2015 (more than 375,000 deaths in absolute terms), corresponding to an estimated 54,000 excess deaths (+9.1%), as compared to the previous season (7), representing the highest reported mortality rate since the Second World War in this country (8). Rapid population growth, climate change, natural disasters, immigration, globalization, and the corresponding sanitation and waste management challenges are expected to intensify the problem in the future (9).

Worryingly, seasonal influenza generally represents an underappreciated public health problem with significant socio-economic implications (10). Monitoring and detecting influenza outbreaks are important but challenging tasks. To accurately track the spread of influenza, reporting systems for influenza-like illness (ILI) and laboratory-confirmed influenza infections (11) can be helpful. These systems are crucial for estimating the number of people experiencing symptoms, hospitalizations, and deaths caused by influenza, addressing vaccination campaigns, and allocating treatment resources. The surveillance of seasonal influenza is possible through data collection and sharing systems, such as FluView in the United States ^1^and FluNews in Europe,^2^ which systematically collect data on seasonal influenza and publish periodic reports to inform on epidemiological trends. Influnet is the Italian nationwide sentinel surveillance system for influenza, coordinated by the Italian National Institute of Health (NIH), collecting epidemiological and virological data that are published weekly on the integrated surveillance system portal ^3^according to an operative protocol^4^ and uploaded into the European database coordinated by the European Centre for Disease Prevention and Control (ECDC) (12). Collaborating sentinel doctors from each region of the country report cases of ILI observed among their patients, collecting, at the same time, biological respiratory samples to identify circulating viruses. The European case definition of ILI was adopted to ensure maximum homogeneity of detection. A case of ILI was defined as a person presenting a sudden and rapid onset of at least one of the following systemic symptoms: fever or feverishness, malaise, headache, myalgia; and at least one of the following respiratory symptoms: cough, sore throat, shortness of breath (13). Doctors take throat swabs from ILI patients tested for influenza viruses at regional Influnet laboratories.

The experience gained over the last few years indicates that the Influenza virus and Coronaviruses are the two main viruses that pose a high risk to humans. Influenza A viruses can infect various animals and humans, leading to pandemics (14, 15). Although environmental virus monitoring can be helpful, the methods are mainly based on clinical data and not validated for environmental testing (16).

Despite this, since the beginning of the COVID-19 pandemic, the utility of wastewater-based epidemiology (WBE) has emerged as a tool for researchers to monitor the circulation of SARS-CoV-2 through the design of pilot studies that highlighted the link between environmental and clinical frameworks (17–22). WBE provides quickly anonymous and aggregated data at a low cost and at a potentially large scale through the passive contributions of the community, therefore integrating the conventional surveillance programs and strengthening health emergency response systems, as occurred with the tracking of the poliovirus during the twentieth century (23). Over the past 2 years, the number of studies supporting wastewater surveillance to monitor the circulation of respiratory pathogens and Influenza viruses in communities has been increasing (9, 24–32). As an effective health assessment approach, WBE has great potential in warning of infectious disease outbreaks for public health (20), as recently demonstrated in Italy during the COVID-19 pandemic (17, 21, 22). Our study aimed to monitor the presence of the influenza virus in the wastewater of different cities on the island. The objective was to evaluate the circulation of the virus throughout an entire Influenza season and compare the results with the conventional integrated epidemiological and virology surveillance.

## 2 Materials and methods

### 2.1 Study design and sample collections

**Figure 1:**
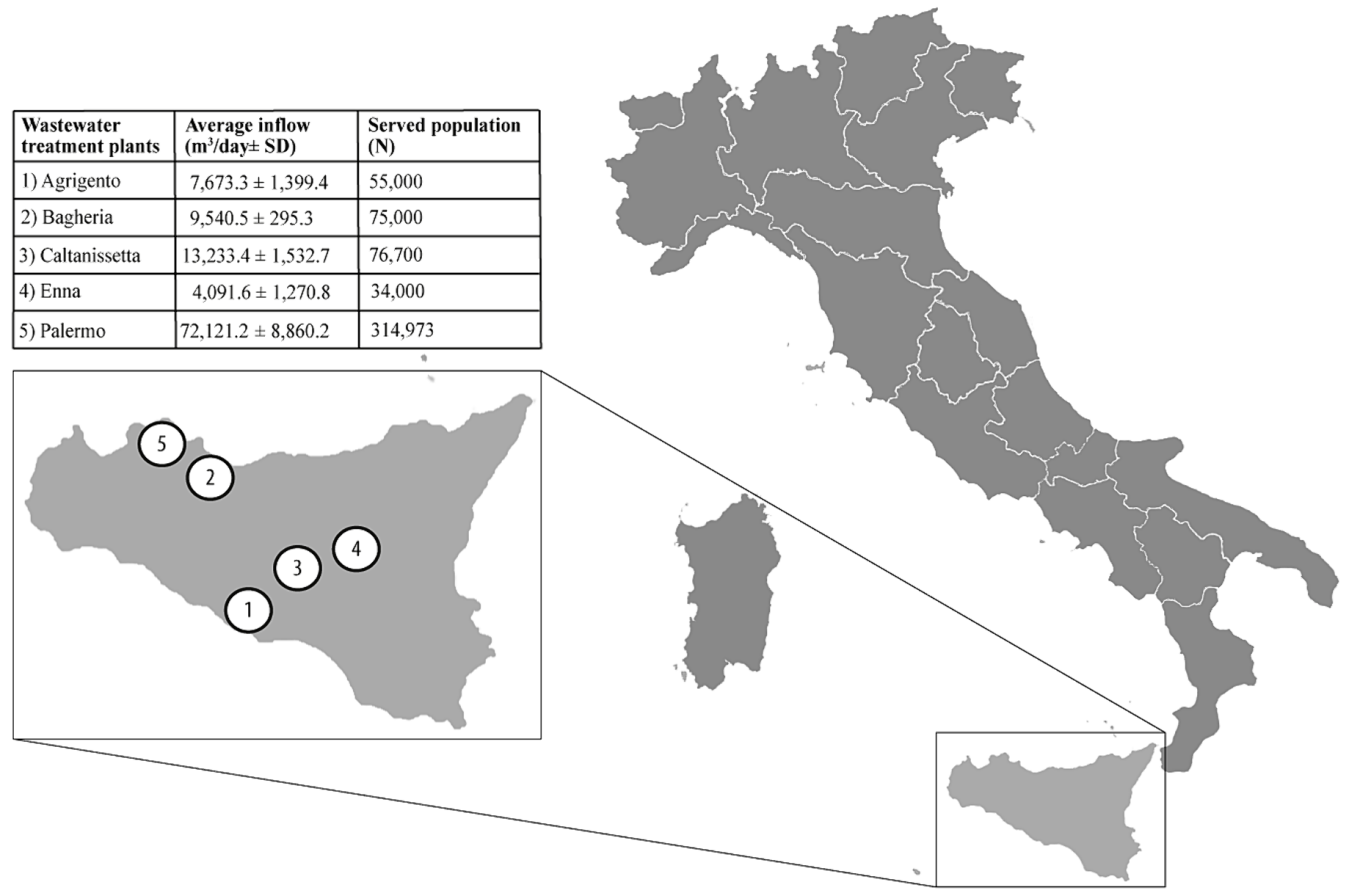
Location and the characteristics of wastewater treatment plant involved in the study.

We conducted an observational study in Sicily (Italy), the largest and most populous island in the Mediterranean Sea, accounting for about 5 million resident inhabitants (33). Five wastewater treatment plants (WTPs) located in five different municipalities, serving a cumulative population of 555,673 inhabitants (ranging from 34,000 to 314,973; 11.1% of total island residents), were included in the study. Raw 24-h composite wastewater samples (*n* = 188) were collected weekly for 9 months, between August 2022 (week 31/2022) and April 2023 (week 17/2023), by an automatic sampling device. Further information about the location and the characteristics of WTPs is provided in Figure 1. The collected samples were refrigerated, transferred to the laboratory, and tested for influenza viral RNA within 24 h from sampling. The wastewater samples collection period (week 31/2022) started before the national epidemiological/virological surveillance (week 42/2022) to assess the viral RNA early detection in wastewater. This evaluation determines if the WBE methodology can serve as an early warning system for influenza circulation.

### 2.2 Virus concentration

All samples underwent a 30-min treatment at 56°C to minimize the potential impact of bioaerosol on personnel and environmental safety (34–37). Heat-treating samples at 56°C for 30 min should cause a negligible or little effect on the sensitivity of RT-PCR (17, 38, 39). Then, each sample was concentrated using a polyethylene glycol (PEG)-based procedure, according to Wu et al. (40) protocol with minor modification. Briefly, wastewater samples (45 mL) were centrifuged at 4,500 x g for 30 min; after centrifugation, 40 mL of sample were mixed with 8% w/v polyethylene glycol 8.000 and 0.3 M NaCl (both supplied by Sigma-Aldrich, St. Louis, MO, USA), spiked with a known amount of Murine Norovirus, used as a process control. After a centrifugation step at 12,000 x g for 2 h, the viral pellet was resuspended in 2 mL of NucliSENS Lysis Buffer reagent (bioMerieux, Marcy-l’Étoile, France) for sub-sequent RNA extraction.

Viral RNA extraction was performed using a semi-automated system based on lysis and magnetic silica beads (supplied by bioMerieux, Marcy l’Etoile, France). After an incubation of 20 min at room temperature, 100 μL of magnetic silica beads were added. After further incubation for 10 min, an automated procedure was performed using the nucleic acid purification system (Auto-Pure96, All Sheng Instruments, Zhejiang, China). Before molecular tests, the extracted nucleic acids in an eluent volume of 100 μL, were purified from potential PCR inhibitors using the OneStep PCR Inhibitor Removal Kit (Zymo Research, CA, USA).

### 2.3 RT-qPCR

One-step real-time reverse-transcription (RT) quantitative PCR assays were used to detect the presence of Influenza A viral RNA (IAV) and/or Influenza B viral RNA (IBV) according to the CDC protocol with minor modifications.^5^ A test was considered positive when its cycle threshold (Ct) value was <40. All q-PCR assays were performed with singleplex real-time PCR (rPCR) assays using the TaqMan technology and run on a QuantStudio™ 7 Flex Real-Time PCR System (Applied Biosystems, Carlsbad, CA, USA); primers, probes sets and reagents are described in Tables 1, 2. For the detection of viral RNA, we performed q-PCR as a single step using the Quantinova Pathogen + IC kit Polymerase (Qiagen, CA, USA). The PCR conditions were as follows: 1 cycle at 50°C for 2 min; 1 cycle at 95°C for 2 min; 45 cycles at 95°C for 15 s and 55°C for 30 s.

**Table 1:** Primers and probes for detecting influenza A, influenza B and Murine Norovirus by q-PCR.

Name	Description	Oligonucleotide sequence (5′–3′)
InfA-F	InfA For1	CAA GAC CAA TCY TGT CAC CTC TGA C
	InfA For2	CAA GAC CAA TYC TGT CAC CTY TGA C
InfA-R	InfA Rev1	GCA TTY TGG ACA AAV CGT CTA CG
	InfA Rev2	GCA TTT TGG ATA AAG CGT CTA CG
InfA-P	InfA Probe	FAM/TGC AGT CCT CGC TCA CTG GGC ACG/BHQ
InfB-F	InfB For	TCC TCA AYT CAC TCT TCG AGC G
InfB-R	InfB Rev	CGG TGC TCT TGA CCA AAT TGG
InfB-P	InfB Probe	FAM/CCA ATT CGA GCA GCT GAA ACT GCG GTG/BHQ
MNV orf1/2junct/F	MNV For	CAC GCC ACC GAT CTG TTC TG
MNV orf1/2junct/R	MNV Rev	GCG CTG CGC CAT CAC TC
MNV orf1/2junct/P	MNV Probe	FAM/CGC TTT GGA ACA ATG/MGBNFQ

**Table 2:** The PCR reagents.

Reagent for flu A detection	Final concentration (nM)	Volume (μl)
Quantinova Master Mix*	–	3.90
InfA For1	400	0.15
InfA For2	400	0.15
InfA Rev1	600	0.225
InfA Rev2	200	0.075
InfA Probe	300	0.45
Nuclease free water	–	5.05
Sample	–	5.00
Total volume	15.00	
Reagent for flu B detection	Final concentration (nM)	Volume (μl)
Quantinova Master Mix*	–	3.90
InfB For	800	0.30
InfB Rev	800	0.30
InfB Probe	300	0.45
Nuclease free water	–	5.05
Sample	–	5.00
Total volume	15.00	
Reagent for Murine Norovirus detection	Final concentration (nM)	Volume (μl)
Quantinova Master Mix*	–	3.90
InfB For	300	0.15
InfB Rev	600	0.30
InfB Probe	200	0.15
Nuclease free water	–	5.50
Sample	–	5.00
Total volume	15.00	

*Quantinova Mastermix is premixed with 15 μL of ROX reference dye before use.

Viral RNA quantification was performed using 10-fold dilutions, ranging from 1.0 to 1.0 × 10^5^ Genomic Copies (GC)/μL per reaction, of a synthetic double-stranded plasmid construct carrying IAV and IBV nucleotide sequences specific for the real-time assays. qPCR standard curves were generated by linear regression of Ct values versus log10 standard concentration and used to convert Ct values into influenza RNA copies/μL per reaction (Slope = − 3.385; *R* = 0.999; Efficiency (%) = 97.422; *Y*-intercept = 21.721). The influenza viral RNA’s GC/L in wastewater was obtained according to the formula: (GC/μL x (100 μL/40 mL)) x 1.000 mL/1 L. The results were also evaluated in GC/day/inhabitant according to the following formula: flow rate of WTP in 24 h (m^3^) x GC (GC/L)/equivalent number of inhabitants served by the WTP. Verification of PCR inhibition was performed as a quality parameter of the determinations. To verify the inhibition, the PCR Ct value obtained from the sample added with 1 μL of a 1.0 × 10^3^ GC/μL of the synthetic double-stranded plasmid was compared with the PCR Ct value of water for molecular biology added with 1 μL of the same synthetic double-stranded plasmid, according to the following formula: ΔCt = Ct (sample + control plasmid) – Ct (water + control plasmid). The sample was considered acceptable if ΔCt was ≤2. Before performing sample analysis, the limit of detection (LoD) was determined by spiking wastewater extracts with dilutions of the synthetic double-stranded plasmid solutions at concentrations of approximately 1,000, 100, 50, 20, 10, 2, and 1.0 GC/μL. Ten replicates of each dilution were tested. The LoD was the lowest concentration at which all ten replicates were positive. The assay had a LoD of 2.5 GC/μL. The concentration/extraction efficiency of the method was assessed as previously reported (22). The sample was considered acceptable if the concentration/extraction efficiency was ≥1%.

### 2.4 Clinical and virologic data sources

We accessed the Influnet web-based platform data (41) to obtain weekly national and regional epidemiological and virological reports, including the ILI incidence per 1,000 inhabitants for the Sicilian region and the aggregate number of influenza-positive swabs. Specifically, data were retrieved from week 42/2022 (conventionally marked as the starting week for influenza virus circulation and thus established as the onset time for the start of the national influenza circulation surveillance-system data collection) up until week 17/2023 (considered as the ending of influenza season).

### 2.5 Statistical analyses

The national surveillance influenza platform contains regional data regarding influenza virus surveillance. Since data collection was performed weekly, IAV and IBV viral loads (intended as viral RNA copy numbers per day/inhabitants of wastewater) detected from the five Sicilian WTPs were aggregated in weekly means and summed, thus obtaining the total IAV + IBV viral load. Moreover, new time-dependent variables (lag times) were created to assess the wastewater detection method’s early-warning capacity. They were based on a method we already performed in our previous WBE study (42). Specifically, by using “WTPs sampling week” and “regional ILI incidence per 1,000 inhabitants” as key variables, the incidence was set at week 0 (intended as the week of sample collection), week 1 and 2 (respectively, 1 and 2 weeks ahead of the WTPs’ sampling week).

As viral concentrations in wastewater are log-normally distributed, a log-10 transformation was applied for all the variables we analysed. Thus, although WBE data were collected from week 31/2022 to assess early virus circulation, national surveillance data were available from week 42/2022. Thus, Person’s correlation test, log-linear regression analyses and significance tests, retrieving R, r^2^ and *p*-values, were carried out through RStudio software (version 4.2.2) to compare from week 42/2022 to week 17/2023, at weeks 0, 1 and 2, the following variables:

- The mean weekly regional ILI incidence per 1,000 inhabitants with the weekly average of combined IAV and IBV viral loads derived from WTPs.
- The weekly regionally combined number of positive IAV and IBV swabs detected, with the combined IAV and IBV Regional viral load detected from WTPs.

The Shapiro–Wilk test was carried out to check for the normality of each continuous variable. A *p*-value <0.05 was considered statistically significant.

## 3 Results

**Figure 2:**
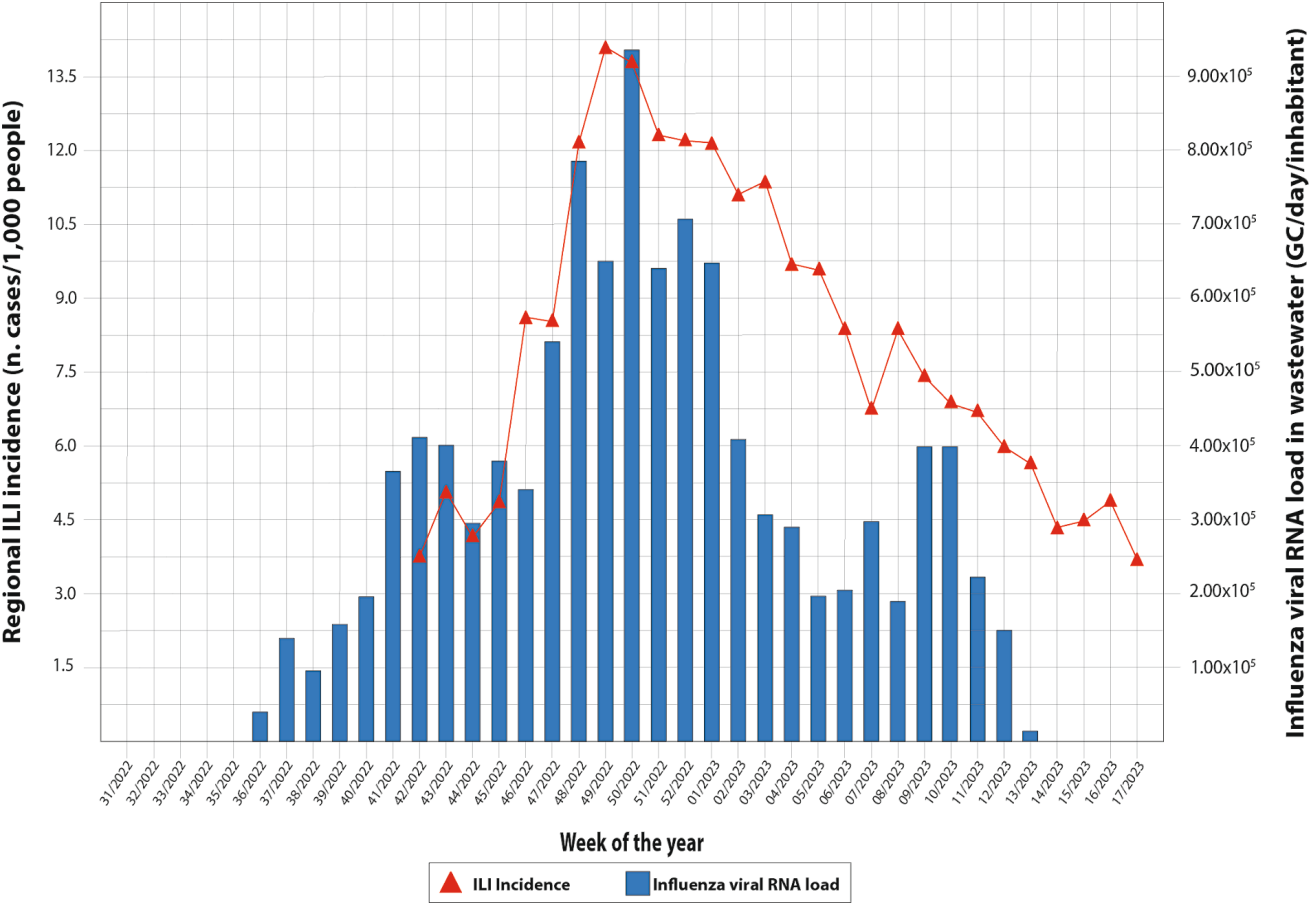
Weekly trends in the ILI regional incidence, reported by the national surveillance system and the influenza virus load in sewage per week of the year.

**Figure 3:**
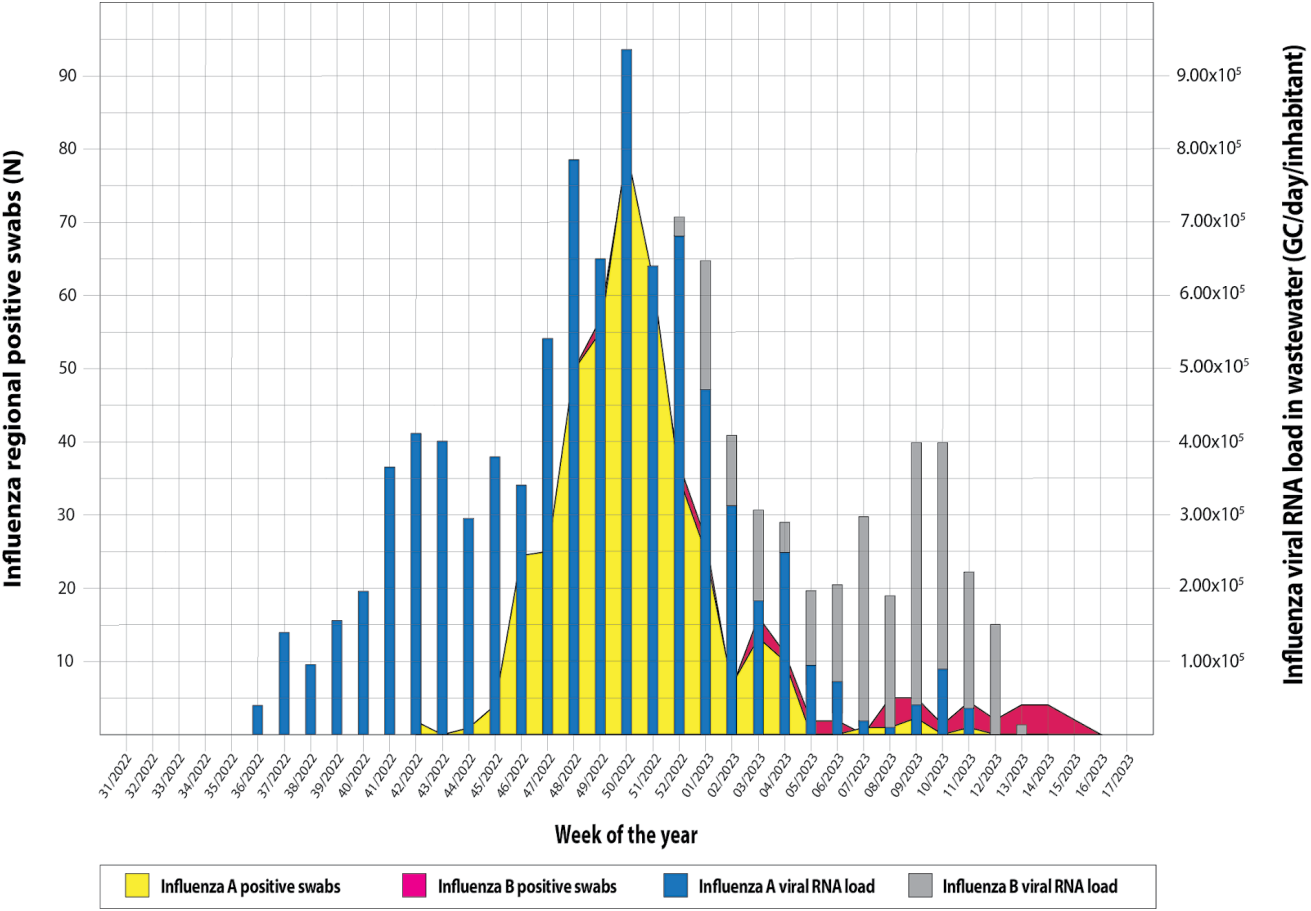
Trend of Influenza virus circulating regionally system and viral load detected from the local wastewater samples.

Overall, from 7 September 2022 to 30 April 2023, 189 wastewater samples were investigated every week. In particular, the following samples were collected from five municipalities and tested for IAV and IBV RNA: Agrigento (*n* = 36), Bagheria (*n* = 37), Caltanissetta (*n* = 39), Enna (*n* = 39), and Palermo (*n* = 37). Overall, IAV RNA was detected in 123/189 samples (65.1%) and IBV RNA in 37/189 samples (19.5%), while the co-presence of the two viral RNA was recorded in 22/189 (11.6%) of the analyzed samples. The recovery rate of influenza viral RNA has ranged from 1 to 100% (mean 8.72; 95% C.I. = 6.35–11.09), compared to a Murine Norovirus control of known concentration in PCR grade water. Table 3 shows the descriptive analysis of the main clinical and virological surveillance data of the flu season 2022/2023. In the entire study period, the concentration of IAV in wastewater ranged from 0.0 to 9.3 × 10^5^ GC/day/inhabitants, while IBV ranged from 0.0 to 3.5 × 10^5^ GC/day/inhabitants. Figure 2 depicts the weekly trends in the ILI regional incidence, reported by the national surveillance system (primary y-axis) and the influenza RNA load in sewage (secondary y-axis) per week of the year (x-axis). In week 36/2022, the first influenza-positive wastewater samples were recorded, with an average concentration of 4.4 × 10^4^ GC/day/inhabitants. In the following weeks, there was a constantly increasing trend of viral RNA detected in the wastewater until reaching the peak of 9.3 × 10^5^ GC/day/inhabitants in week 50/2022. From then on, the viral RNA concentration in wastewater progressively decreased until week 06/2023, after which a second lower peak occurred at week 10/2023, quantified as 3.9 × 10^3^ GC/day/inhabitants. After that, the viral RNA concentration in wastewater regularly decreased until the absence of detection from week 14/2023. On the other hand, the epidemiological trend of ILI at a regional level showed high values starting from week 42/2022, the first surveillance week of the 2022/2023 season, and peaked in week 49/2022. Excluding small occasional increases in ILIs, the trend has been downward until the end of the surveillance season scheduled for week 17/2023. The number of cumulative ILI cases substantially overlapped with the influenza RNA load from wastewater samples, with an increasing trend of influenza viral RNA in wastewater samples comparable to the rise of ILI cases in the population. Figure 3 shows the trend of IAV and IBV circulating regionally, obtained from the virological surveillance system and the viral RNA load detected from the local wastewater samples. The wastewater analyses allowed us to record the total presence of IAV from week 36/2022 until week 51/2022. From week 52/2022 and up to week 13/2023, there was a co-circulation of the two types of viruses, and the concentration of IBV had an increasing trend until its peak recorded at week 09/2023 with a concentration of 3.5 × 10^5^ GC/day/inhabitants. In confirmation of the co-circulation of viruses from week 52/2022 and of the subsequent predominance of IBV over IAV from week 05/2022, the ratio of IBV over IAV showed values of 0.1 in week 52/2022, of 1.5 in week 05/2023 and 12.8 in week 07/2022 and, in any case, always greater than one up to week 11/2023, the last in which the wastewater samples gave a positive result. A similar trend was shown by the regional virological surveillance of influenza-positive swabs, in which from week 46/2022 to week 50/2022, there was an exclusive circulation of the IAV, a co-circulation of both viruses up to week 17/2023 with a predominance of IBV from week 06/2023 to week 17/2023, with a ratio of type B to type A ranging from 1.2 to 4.8.

**Table 3:** Descriptive analysis containing the total weekly mean Influenza virus load assessed in wastewater from the different WTPs, the regional weekly ILI incidence per 1,000 inhabitants, the total number of regional swabs performed and the positivity rate.

Week	Regional ILI incidence (x 1,000 inhabitants)	Total swabs performed (*N*)	Positivity rate (%)	Viral load (GC/day/inhabitants)
42/2022	3.7	20	10.0	4.1 × 10^5^
43/2022	4.8	2	0.0	4.0 × 10^5^
44/2022	4.0	1	100.0	2.9 × 10^5^
45/2022	4.7	8	50.0	3.8 × 10^5^
46/2022	8.7	63	38.0	3.4 × 10^5^
47/2022	8.6	75	33.3	5.4 × 10^5^
48/2022	12.2	92	53,3	7.8 × 10^5^
49/2022	14.0	102	54.0	6.5 × 10^5^
50/2022	13.6	135	58.5	9.3 × 10^5^
51/2022	12.3	130	47.7	6.3 × 10^5^
52/2022	12.1	99	37.3	7.0 × 10^5^
01/2023	12.0	100	25.0	6.5 × 10^5^
02/2023	10.9	119	5.9	4.1 × 10^5^
03/2023	11.9	85	15.3	3.1 × 10^5^
04/2023	9.6	51	19.6	2.8 × 10^5^
05/2023	9.5	34	34.0	1.8 × 10^5^
06/2023	8.1	29	29.0	2.0 × 10^5^
07/2023	6.2	13	13.0	2.9 × 10^5^
08/2023	8.2	30	16.6	1.8 × 10^5^
09/2023	7.5	16	31.2	3.9 × 10^5^
10/2023	7.2	13	13.0	3.9 × 10^5^
11/2023	6.7	25	16.0	2.2 × 10^5^
12/2023	6.0	21	9.5	1.5 × 10^5^
13/2023	5.6	17	17.6	1.7 × 10^4^
14/2023	4.3	5	60.0	0.0
15/2023	4.5	10	20.0	0.0
16/2023	4.8	1	0.0	0.0
17/2023	3.7	2	0.0	0.0

**Figure 4:**
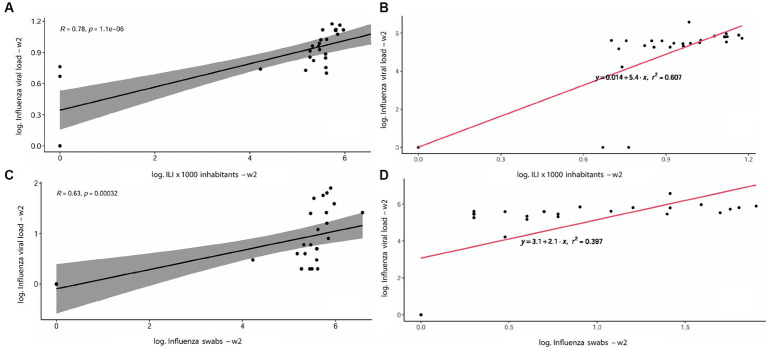
Scatterplots describing the correlation between the RNA viral load detected in wastewater (GC/day/inhabitants) and the number of ILI detected per 1,000 inhabitants at week 2 showing R and *p*-value **(A)** and *r*^2^ **(B)**; the correlation between Influenza viral load in wastewater and the number of positive swabs detected, with *R* and *p*-value **(C)** and *r*^2^ **(D)**.

As shown in Table 3, the correlation analyses between the Influenza viral RNA load (IAV + IBV RNA concentration) detected in WTPs and the regional incidence of ILI per 1,000 inhabitants displayed a *p*-value *< 0.001* at week 0 and *< 0.0001* for weeks 1 and 2, respectively. A moderate-high correlation index (R) was retrieved, ranging from 0.55 at week 0 to 0.78 at week 2. Accordingly, a moderate-correlation index was retrieved when comparing the IAV + IBV viral RNA load detected from WTPs with total number of positive IAV + IBV regionally detected swabs at all times evaluated (Table 4: w0 R = 0.46, *p*-value < 0.01; w1 *R* = 0.55, *p*-value < 0.01; w2 *R* = 0.63, *p*-value < 0.001). In Figure 4 are showed the scatterplots describing the correlation between the RNA viral load detected in wastewater (GC/day/inhabitants) and the number of ILI detected per 1,000 inhabitants at week 2.

**Table 4:** Correlation analysis between the mean weekly RNA viral load in wastewaters and, respectively, the weekly incidence of regional ILI x 1,000 inhabitants in Sicily and the cumulative number of IAV + IBV positive swabs detected in the region at weeks 0, 1 and 2.

	Time	*R*	*r* ^2^	*p*-value
ILI x 1,000 inhabitants (regional) x IAV + IBV GC/day/inhabitant	w0	0.55	0.30	<0.01
	w1	0.70	0.47	<0.0001
	w2	0.78	0.61	<0.0001
*n*°of IAV + IBV positive swabs (regional) x IAV + IBV GC/day/inhabitant	w0	0.46	0.21	<0.01
	w1	0.55	0.30	<0.01
	w2	0.63	0.40	<0.001

## 4 Discussion

Regardless of the influenza season’s onset timing, we observed a rapid and early start of the epidemic season in our study. This resulted in the ample virus circulating in the population when the epidemiological surveillance of the Influnet network began. This trend was also observed in the southern hemisphere, where the Australian data collection systems showed an extremely accelerated and anticipated growth concerning the normal trend (43). In Sicily (Italy), during the first week of surveillance (42/2022, 17–23 October 2022), the incidence of reported ILI, which in principle can be considered a good proxy of the incidence of flu illness (44), was 3.7 cases/1,000 inhabitants, unlike previous influenza seasons which stood at decidedly lower values (41). The anticipated presence of the circulation of influenza viruses was also recorded through the analysis of wastewater, which began in the week of 31/2022 (1–7 August 2022). In week 36/2022 (5–11 September), we simultaneously detected influenza viruses in all municipalities through wastewater analysis. This was 17 days before the start of conventional national surveillance. The values recorded ranged from 6.00 × 10^2^ to 1.24 × 10^3^ GC/L. Unfortunately, we cannot determine the specificity of our method due to the unavailability of sufficient clinical swabs from sentinel doctors for each municipality. Nonetheless, this early detection of pathogen circulation through WBE has the potential to benefit public health greatly. It could aid in differentiated programming of the start of epidemiological/virological surveillance and vaccination campaigns to increase their effectiveness.

A sustained co-circulation of type A and B influenza viruses characterized Italy’s 2022/2023 influenza season. Overall, IAV was prevalent (79.5% of the samples tested positive) compared to IBV (20.5%). The epidemiological data of influenza that have emerged in the southern hemisphere have attested that influenza has been spreading significantly, probably due to the reduction of distancing measures and the use of masks (43). In the five municipalities in the study, wastewater analyses showed that the majority of IAV was detected in week 50/2022 (12–18 September 2022) with 9.3 × 10^5^ GC/day/inhabitants, while the majority of IBV was found in week 09/2023 (27 February – 05 March 2023) with 3.5 × 10^5^ GC/day/inhabitants. The same trend, with a time lag of 7–14 days concerning wastewater, was recorded by the virological surveillance, which dated the peak circulation of the IAV in the week 49/2022, therefore 7 days earlier, and that of the IBV in week 12/2023, then 14 days later (41).

Our findings confirmed that wastewater surveillance can effectively detect influenza virus circulation and should be considered a valuable supplement to conventional influenza surveillance. More in-depth, it may be used to test influenza virus circulation in the communities for prolonged periods using a single sample approach, like the application of SARS-CoV-2 WBE used to monitor the prevalence of COVID-19. The WBE methodology could be an integrative approach to epidemiological and virological surveillance that introduces some interesting aspects to improve the estimation of influenza incidence. By monitoring various treatment plants in the city, the percentage of subjects tested can be increased compared to the Virological Surveillance Network’s target of 4% of the regional population. Additionally, collecting and transporting wastewater is more straightforward, cheaper, and potentially feasible wherever there is a sewage network, thus increasing the possibility of obtaining information even in smaller municipalities that are typically excluded from traditional surveillance systems. While there are many advantages to infectious disease wastewater monitoring, the WBE approach has some limitations, including aggregated data and the inability to perform epidemiological assessments by age groups, symptoms, or immune status for vaccinated subjects. Wastewater is a complex matrix affected by environmental factors that are not always identified, leading to inherent variability and uncertainties (45, 46). Furthermore, it’s important to address the lack of standardized protocols in the various phases of the analytical process. This includes sample pre-treatment, concentration, and nucleic acid detection (47). We need to establish a testing framework that considers the different analytical sensitivities at each analysis step. For example, in the thermal pretreatment phase, some studies show negligible changes in RNA measurement (34–37), while others do not (48–50). Similarly, in the concentration phase, the PEG-supernatant may have limitations due to the nature of influenza viruses, which have an envelope. This means it may not be suitable as the reference sample for conducting an influenza-WBE study, despite successful use in other studies globally (26, 51). The direct consequence is the difficulty of determining how directly wastewater concentrations reflect the number of infected individuals (28).

Wastewater-based methods can provide insight into the circulation of respiratory viruses within a specific community without testing numerous individuals. This is because a single wastewater sample represents the entire community’s contribution. The results from wastewater testing can be obtained within 24 h of sample collection, providing real-time information that can be used to inform public health responses, clinical decision-making, and individual behavior modifications.

## Data availability statement

The datasets presented in this study can be found in online repositories. The names of the repository/repositories and accession number(s) can be found at: https://www.salute.gov.it/portale/influenza/dettaglioContenutiInfluenza.jsp?lingua=italiano&id=704&area=influenza&menu=vuoto&tab=5.

## Ethics statement

Ethical approval was not required for the studies involving humans because the information collected is aggregated and anonymous. The studies were conducted in accordance with the local legislation and institutional requirements. The human samples used in this study were collected anonymously and in an aggregated way at wastewater purification plants. No information on humans was collected. Written informed consent to participate in this study was not required from the participants or the participants' legal guardians/next of kin in accordance with the national legislation and the institutional requirements.

## Author contributions

CM: Conceptualization, Funding acquisition, Methodology, Validation, Writing – original draft. WM: Supervision, Writing – original draft. WP: Data curation, Formal analysis, Visualization, Writing – review & editing. RP: Data curation, Formal analysis, Visualization, Writing – review & editing. GG: Data curation, Formal analysis, Visualization, Writing – review & editing. CC: Data curation, Formal analysis, Visualization, Writing – review & editing. AR: Investigation, Resources, Writing – review & editing. GA: Investigation, Resources, Writing – review & editing. IR: Investigation, Resources, Writing – review & editing. VGi: Investigation, Resources, Writing – review & editing. FI: Investigation, Resources, Writing – review & editing. AS: Investigation, Resources, Writing – review & editing. RL: Investigation, Resources, Writing – review & editing. VGu: Investigation, Resources, Writing – review & editing. FV: Supervision, Writing – review & editing. FT: Conceptualization, Methodology, Writing – original draft.

## Footnotes

^1^ www.cdc.gov/flu/weekly

^2^ www.flunewseurope.org

^3^ https://respivirnet.iss.it/pagine/rapportoInflunet.aspx

^4^ https://www.salute.gov.it/imgs/C_17_pubblicazioni_3267_allegato.pdf

^5^ https://www.cdc.gov/coronavirus/2019-ncov/lab/multiplex.html
